# HIV Protease Inhibitors Do Not Cause the Accumulation of Prelamin A in PBMCs from Patients Receiving First Line Therapy: The ANRS EP45 “Aging” Study

**DOI:** 10.1371/journal.pone.0053035

**Published:** 2012-12-28

**Authors:** Sophie Perrin, Jonathan Cremer, Olivia Faucher, Jacques Reynes, Pierre Dellamonica, Joëlle Micallef, Caroline Solas, Bruno Lacarelle, Charlotte Stretti, Elise Kaspi, Andrée Robaglia-Schlupp, Corine Nicolino-Brunet Catherine Tamalet, Nicolas Lévy, Isabelle Poizot-Martin, Pierre Cau, Patrice Roll

**Affiliations:** 1 Inserm UMR_S 910, Aix-Marseille Université, Marseille, France; 2 Laboratoire de Biologie Cellulaire, Centre Hospitalier Universitaire (CHU) La Timone Assistance Publique des Hôpitaux de Marseille (APHM), Marseille, France; 3 Service d’Immuno-Hématologie Clinique, Centre Hospitalier Universitaire (CHU) Sainte Marguerite Assistance Publique des Hôpitaux de Marseille (APHM), Marseille, France; 4 Département des Maladies Infectieuses et Tropicales, Centre Hospitalier Régional et Universitaire (CHRU) Gui-de-Chauliac, Montpellier, France; 5 Service d’Infectiologie, Centre Hospitalier Universitaire (CHU) L’Archet 1, Sophia-Antipolis Université, Nice, France; 6 Centre d’Investigation Clinique - Unité de Pharmacologie Clinique et d’Evaluations Thérapeutiques (CIC-UPCET), Centre Hospitalier Universitaire (CHU) La Timone Assistance Publique des Hôpitaux de Marseille (APHM), Marseille, France; 7 Laboratoire de Pharmacocinétique et de Toxicologie, Centre Hospitalier Universitaire (CHU) La Timone Assistance Publique des Hôpitaux de Marseille (APHM), Marseille, France; 8 Inserm UMR_S 911, Aix-Marseille Université, Marseille, France; 9 Laboratoire d’Hématologie, Centre Hospitalier Universitaire (CHU) La Conception Assistance Publique des Hôpitaux de Marseille (APHM), Marseille, France; 10 Fédération de Microbiologie Clinique, Centre Hospitalier Universitaire (CHU) La Timone Assistance Publique Hôpitaux de Marseille (APHM), Marseille, France; 11 CNRS (Centre Natonal de la Recherche Scientifique) IRD (Institut de Recherche pour le development) UM 63, Aix-Marseille Université, Marseille, France; 12 Laboratoire de Génetique Moléculaire, Centre Hospitalier Universitaire (CHU) La Timone Assistance Publique des Hôpitaux de Marseille (APHM), Marseille, France; University of Cape Town, South Africa

## Abstract

**Background:**

The ANRS EP45 “Aging” study investigates the cellular mechanisms involved in the accelerated aging of HIV-1 infected and treated patients. The present report focuses on lamin A processing, a pathway known to be altered in systemic genetic progeroid syndromes.

**Methods:**

35 HIV-1 infected patients being treated with first line antiretroviral therapy (ART, mean duration at inclusion: 2.7±1.3 years) containing boosted protease inhibitors (PI/r) (comprising lopinavir/ritonavir in 65% of patients) were recruited together with 49 seronegative age- and sex-matched control subjects (http://clinicaltrials.gov/, NCT01038999). In more than 88% of patients, the viral load was <40 copies/ml and the CD4+ cell count was >500/mm^3^. Prelamin A processing in peripheral blood mononuclear cells (PBMCs) from patients and controls was analysed by western blotting at inclusion. PBMCs from patients were also investigated at 12 and 24 months after enrolment in the study. PBMCs from healthy controls were also incubated with boosted lopinavir in culture medium containing various concentrations of proteins (4 to 80 g/L).

**Results:**

Lamin A precursor was not observed in cohort patient PBMC regardless of the PI/r used, the dose and the plasma concentration. Prelamin A was detected in PBMC incubated in culture medium containing a low protein concentration (4 g/L) but not in plasma (60–80 g/L) or in medium supplemented with BSA (40 g/L), both of which contain a high protein concentration.

**Conclusions:**

Prelamin A processing abnormalities were not observed in PBMCs from patients under the PI/r first line regimen. Therefore, PI/r do not appear to contribute to lamin A-related aging in PBMCs. In cultured PBMCs from healthy donors, prelamin A processing abnormalities were only observed when the protein concentration in the culture medium was low, thus increasing the amount of PI available to enter cells.

ClinicalTrials.gov NCT01038999 http://clinicaltrials.gov/ct2/show/NCT01038999.

## Introduction

The A-type lamins are type V intermediate filaments that are key components of the nuclear matrix. Lamins A and C, which are obtained by alternative splicing of the *LMNA* gene, are the predominant A-type lamins [1]. Lamin C is produced directly as a mature protein, while lamins A undergo several posttranslational modifications [2], [3], [4]. Mature proteins are obtained from their precursors through 4 posttranslational steps. The cysteine residue in the C-terminal CaaX box is farnesylated in the first step [5], following which the aaX residues are removed by the endopeptidases, FACE1/ZMPSTE24 (farnesyl converting enzyme 1/zinc metalloprotease related to yeast ste24p) and/or FACE2/RCE1 (farnesyl converting enzyme 2/Ras-converting enzyme 1) [6]. The cysteine is subsequently carboxymethylated by ICMT (isoprenylcysteine carboxyl methyltransferase) [6]. Following this step, the last 15 C-terminal amino acids of prelamin A are cleaved by FACE1/ZMPSTE24 to release a mature unfarnesylated soluble protein present in both the *lamina* and in the rest of the nucleoplasm [7], [8].

In the *lamina*, lamins interact with several nuclear envelope or nucleoplasmic partners [2], [3], [4], [9]. In addition to their role in determining nuclear shape and size, A-type lamins are believed to form a scaffold that supports a wide variety of mechanisms, including chromatin organization, gene transcription, DNA synthesis and damage repair, cell proliferation and differentiation [2], [3], [4], [9].

Increasing numbers of mutations in *LMNA*, *ZMPSTE24* or genes encoding lamin partners have been associated with tissue-restricted or systemic disorders collectively termed “laminopathies”. Indeed, pathologies of striated muscle [10], [11], [12], peripheral nerve [13] and adipose tissue [14], [15], [16] have been reported. Systemic progeroid syndromes that mimic the clinical features of physiological aging have also been described. The Hutchinson-Gilford progeria syndrome (HGPS) [13], [17] occurs, in most cases, through the *de novo* dominant *LMNA* p.G608G mutation, which results in the persistence of a truncated farnesylated prelamin A (lamin AΔ50, also called progerin) [18]. At clinical levels, these abnormalities lead to severe growth retardation, skeletal alterations (osteolysis, osteoporosis), marked amyotrophy, lipodystrophy and atherosclerosis [18], [19]. Restrictive dermopathy (RD), another systemic progeroid syndrome that is lethal during the perinatal period, is linked to mutations mainly in the *ZMPSTE24* or *LMNA* genes which also result in the persistence of farnesylated prelamin A [20], [21]. Interestingly, low levels of lamin AΔ50 [22] and farnesylated prelamin A (resulting from the FACE1/ZMPSTE24 defect) [23] are produced during physiological aging.

Both treated and untreated HIV (human immunodeficiency virus) infected patients exhibit clinical and biological disorders similar to those observed in genetic laminopathies. Indeed, lipodystrophy, cardiovascular disease, sarcopenia and metabolic abnormalities have been reported in addition to bone and kidney complications [24], [25]. Lipodystrophy has been frequently reported as a side effect of treatment with PIs (protease inhibitors) or with NRTIs (nucleoside reverse transcriptase inhibitors) [26]. Some of these age-related disorders were found to be more common in HIV infected and treated patients than in the general population [27], suggesting a modified time course of aging in HIV patients.

Alterations in lamins have previously been reported during viral infection (herpes simplex virus [28]; cytomegalovirus [29]). During HIV-1 infection, Vpr (viral protein r) induced a transient local disassembly of *lamina* that resulted in nucleoplasm herniations [30]. This mechanism is believed to allow the penetration of HIV pre-integration complex into the nucleoplasm. Interestingly, similar but permanent herniations were found in HGPS or RD cells, and represent a classical feature of genetic laminopathies [20], [21]. Besides the transient effect of HIV proteins, *lamina* alteration could also be due to treatment with boosted protease inhibitors (PI/r).

Indeed, in cultured cells, some PIs have been reported to inhibit FACE1/ZMPSTE24 [31], [32] and induce the persistence of farnesylated prelamin A [31], [32], [33], [34]. Reduction of the lamin A mRNA level has also been highlighted in adipose tissue from HIV infected and treated patients regardless of the clinical presence of lipodystrophy [35]. Prelamin A has been detected in adipose tissue from lipodystrophic HIV infected patients treated with first generation PIs (indinavir or nelfinavir) [36], [37]. In spite of these observations, the role of lamin A in the aging of HIV infected and treated patients remains to be clarified.

The ANRS EP45 “Aging” study investigates the cellular mechanisms involved in aging in these patients. Functional and morphological parameters in mitochondria, organelles known to be involved in cellular aging, have already been explored in peripheral blood mononuclear cells (PBMCs) from our cohort [38]. The present study focused on prelamin A in PBMCs from 35 HIV-1 infected patients treated with first line antiretroviral therapy (ART) comprising 2NRTI+1PI/r. No prelamin A was detected at inclusion or up to two years after enrolment in the study, regardless of the PI/r used, their dose or their plasma concentration. PI/r do not appear to contribute to lamin A-related aging in PBMCs. Moreover, we demonstrated that prelamin A could be detected in PBMCs incubated with PI/r, but only if the culture medium contained a low protein concentration, at least ten times less than in human plasma. Our results may explain the discrepancy between the detection of prelamin A in cultured cells and patient peripheral blood cells.

## Methods

### ANRS EP45 Study & Participant Characteristics

The ANRS EP45 “Aging” study is a cross-sectional and longitudinal multicentric study (Marseille, Montpellier and Nice; France). The protocols for this trial is available as supporting information, see Protocol S1. Basic demographic, clinical, biological parameters and ART combinations are partially detailed in Tables S1 and S2 from [38].

Briefly, 179 participants were enrolled according their HIV-1 or ART status. Among them, 35 HIV-1 infected patients receiving first line ART comprising a 2NRTI and PI/r regimen for at least 12 months were recruited at inclusion (M0; Month 0). PIs were administered twice or once daily according clinician’s prescription. Atazanavir was exclusively administered once daily. Lopinavir and amprenavir were administered twice daily in 90% and 80% of patients respectively. At inclusion, the mean treatment duration was 2.7 (±1.3) years. The PI/r regimen contained lopinavir (69% of patients), atazanavir (14%) or fosamprenavir (14%). Ritonavir was always used as a booster. None of the patients were treated with an ART regimen containing boosted darunavir. Forty-nine seronegative age- and sex-matched subjects were used as a control group. Patients were followed-up one (M12; month 12) and two years (M24; month 24) after inclusion (M0).

We report here the nuclear protein data measured at M0, M12 and M24.

### Ethics Statement

The French Health Products Safety Agency Regulatory Authority (AFSSAPS, Agence Française de Sécurité Sanitaire des Produits de Santé) and Marseille Ethical Committee (Comité de Protection des Personne Sud Méditerranée I) approved the protocol. The study, registered on the ClinicalTrials.gov web site (Identifier: NCT01038999), was performed in accordance with the Declaration of Helsinki. All subjects gave written informed consent before participation.

### Blood Puncture

Blood samples from patients under treatment were simultaneously collected in EDTA (for cellular analyses) and in heparin-lithium (for PI/r plasma concentration assays).

### PI/r Plasma Concentration

Lopinavir remained stable during our sample processing (data not shown) in accordance with the well-known stability of PIs [39], [40], [41], [42], [43]. Plasma separation was performed on receipt of the blood tubes, following which the plasma was stored at −20°C prior to analysis. The concentration of PI/r in plasma was quantified by analysing plasma recovered from blood collected in lithium heparinized tubes (BD Vacutainer) using a specific and sensitive validated liquid chromatography-tandem mass spectrometry (LC-MS/MS) method [44].

### PBMC Isolation from Patients

To standardize sample processing despite shipping delays between HIV Clinical Units (Montpellier, Nice and Marseille) and the Cell Biology Laboratory (Marseille), blood collected in EDTA Vacutainer tubes (Becton Dickinson) was rotated overnight at room temperature before PBMCs isolation using Ficoll®. Cells viability was maintained at >97% (data not shown). PBMCs were isolated by Ficoll® gradient centrifugation (UNI-SEP MAXI+, Novamed) according to the manufacturer’s instructions. Leucocyte formulae were calculated in May-Grünwald-Giemsa-stained Cytospin® samples. Directly after PBMC isolation, residual red blood cells, if any, were hypotonically lysed in 100 µM EDTA, 150 mM NH_4_Cl, 1 mM KHCO_3_, pH 7.4. Cells were stored immediately at −80°C.

### Reagents

Lopinavir was kindly provided by Abbot to the Department of Pharmacokinetics and Toxicology (APHM Timone Hospital, Marseille, France). A 20 mM stock solution was prepared in DMSO.

**Zo**ledronic acid (Zometa®, Novartis) and sodium **pra**vastatine (Phr.Eu quality, Hisun Pharmaceuticals) were used in combination (ZoPra). Stock solutions were 2.76 mM for zoledronate (diluted in H_2_O) and 8.96 mM for pravastatine (diluted in PBS).

### Incubation of PBMCs from Healthy Donors with Lopinavir/r

Freshly isolated PBMCs from healthy donors were incubated in RPMI (Life Technologies) containing 10% FBS (foetal bovine serum; Life Technologies) and 2 mM L-glutamine (Life Technologies) for 24 hours at 37°C in a humidified atmosphere containing 5% CO_2_. PBMCs were treated with lopinavir (concentration range: 2, 20, 40 and 200 µM). PBMCs were also incubated with the same concentration of lopinavir in medium containing lopinavir at the same concentrations that was also supplemented with bovine serum albumin (BSA) up to 40 g/L or plasma recovered during PBMCs isolation. PBMCs incubated with DMSO only (≤1% depending of the PI concentration used) were used as negative control cells. Experiments were performed at least in triplicate.

Cell viability was determined using 0.4% trypan blue (Lonza). At least 200 cells were counted per condition.

### Incubation of PBMCs from Healthy Donors with ZoPra

The ZoPra combination blocks the farnesylation of prelamin A [45], thus preventing the cleavage of prelamin A by FACE1/ZMPSTE24 to generate mature lamin A. In control conditions except during physiological aging [22], [23], normal maturation of lamin A precludes the detection of its prelamin A precursor. Therefore, we used the ZoPra combination to block prelamin A processing in PBMCs such that prelamin A accumulated in the cells. PBMCs from healthy seronegative subjects were incubated in culture medium containing 60 µM of ZoPra for 24h at 37°C, 5% CO_2_. These cells were used as a positive control for the detection of prelamin A by western blotting (WB), immunofluorescence (IF) and flow cytometry analysis (Figure S1).

### Protein Extraction

Total PBMC proteins were extracted in 2X Laemmli buffer (Tris base pH 6.8, 6% SDS, 10% β-mercaptoethanol, 10% glycerol, 0,1 M dithiothreitol [34]).

### Western Blotting

Protein lysates were separated on Nupage® Novex 8% Bis-Tris precast gels (Life Technologies) and transferred to Immobilon-FL PVDF membranes (Millipore). Membranes were blocked for one hour in 1∶2 diluted blocking buffer for near infrared fluorescent western blotting (Rockland). Blocked membranes were incubated with primary antibodies for one hour at room temperature (RT), following which they were washed and incubated with IR-Dye conjugated secondary antibodies for one hour at RT. Bound antibodies were detected and analysed on an Odyssey® V3.0 imaging system (LI-COR Biosciences) according to the manufacturer’s instructions. Glyceraldehyde 3-phosphate dehydrogenase (GAPDH) was used as a total cellular protein loading control.

### Immunofluorescence

Immunofluorescence staining of lamin A and prelamin A was performed in PBMC from 179 cohort participants at M0 according to a standard procedure, as described in Methods S1. Experiments were performed blinded with respect to subject status (treated HIV-1 patients or control subjects).

### Antibodies

The following antibodies were used in this study: mouse monoclonal anti-lamin A/C (MAB3211, 1/200, Millipore), goat polyclonal anti-lamin A/C (sc6215, 1/200, Santa Cruz), rabbit polyclonal anti-lamin A/C (sc20681, 1/200, Santa Cruz), goat polyclonal anti-prelamin A (sc6214, 1/200, Santa Cruz), rabbit polyclonal anti-prelamin A (ANT0045, 1/100, Diatheva), mouse monoclonal anti-GAPDH (MAB374, 1/40,000, Millipore).

Secondary antibodies conjugated with IR-Dye 800CW or 680 were used according to the manufacturer’s instructions (926-32212, 926-32214, 926-32223, 926-32224, 1/5000, LI-COR® Biosciences).

### Statistical Analysis

Statistical and box plot analyses were performed using GraphPad Prism 5.04 (GraphPad Software, Inc.). Cell viability was compared between conditions using the Mann-Whitney test. p values of <0.05 were considered statistically significant. Box plots of the PI plasma concentration from patients displayed the median, min and max values.

## Results

### ANRS EP45 “Aging” Patients Exhibited Good Compliance to Treatments and Mainly Remained in the Common Drug Therapeutic Range

The total plasma PI/r level, which includes both bound and unbound drug, was evaluated for each patient at each follow-up visit. Samples were drawn 10–12 hours post-dose (for twice daily administration) or 20–24 hours post-dose (for once daily administration) to obtain the trough concentration (Cmin). In some cases, samples were drawn at the maximal concentration (Cmax) (3–6 hours, 2–4 hours and 1–3 hours after drug intake for lopinavir, atazanavir and fosamprenavir, respectively).

**Figure 1:**
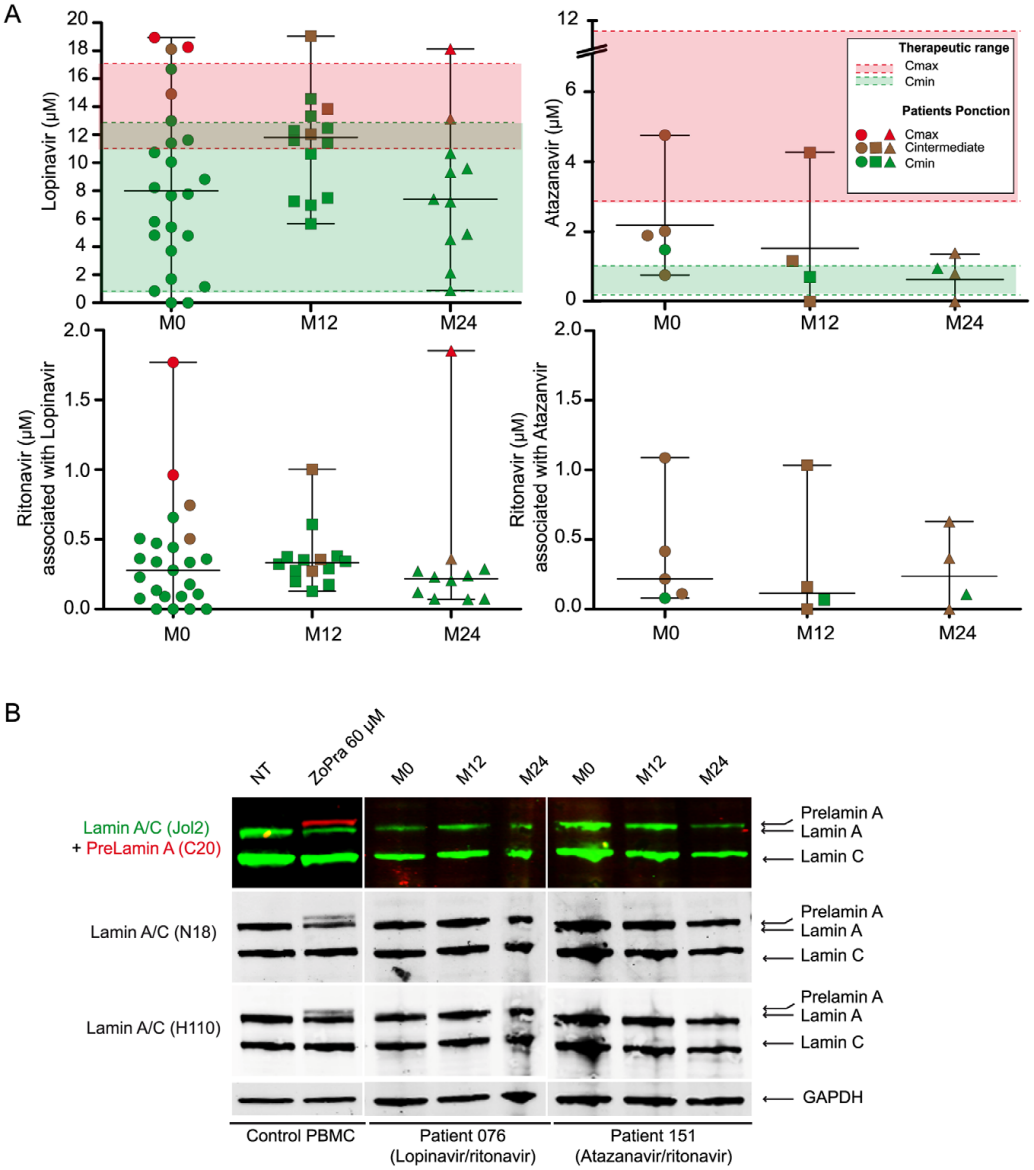
Lack of prelamin A in PBMCs from compliant patients receiving the 2NRTI+1PI/r regimen. (**A)** Plasma concentrations of lopinavir, atazanavir and ritonavir from ANRS EP45 “Aging” patients reported at M0 (circles), M12 (square) and M24 (triangle). Colors indicate the durations between last drug uptake and venipuncture (Cmax, red; Cmin, green, Cintermediate, brown) and PI therapeutic concentration range (Cmax, red; Cmin, green). The majority of patients receiving lopinavir treatment were assayed at Cmin and remained within the therapeutic range. Samples from patients being treated with the atazanavir regimen were mainly assayed at mid-dose. (**B**) Representative western blots of PBMCs from patients receiving lopinavir or atazanavir treatment using three different lamin A/C-specific antibodies and one prelamin A-specific antibody. No prelamin A was detected in PBMCs from patients. Control PBMCs from healthy controls incubated with ZoPra were used as positive control cells. PI plasma concentrations in the patients shown: lopinavir 10.7 µM (M0), 12.3 µM (M12) and 9.3 µM (M24); atazanavir 1.5 µM (M0), 0.7 µM (M12) and 0 µM (M24).

The lopinavir median plasma concentrations were 8 µM (range: 0–18.9) at M0, 11.8 µM (5.9–19) at M12 and 7.4 µM (0.8–18.1) at M24 (Figure 1A), which corresponded to Cmin or Cmax in 82–85% and 8–9% of cases, respectively. Of the lopinavir plasma samples from our patients, 70% (M0), 69% (M12) and 82% (M24) of lopinavir plasma samples from our patients remained within the recommended therapeutic range (Cmin, 1.6–12.7 µM; Cmax, 11.1–17.4 µM). The corresponding ritonavir median plasma concentrations were 0.28 µM (range: 0–1.8) at M0, 0.33 µM (0.13–1.0) at M12 and 0.22 µM (0.07–1.8) at M24 (Figure 1A).

The atazanavir median plasma concentrations were 1.9 µM (range: 0.7–4.8) at M0, 0.9 µM (0–4.3) at M12 and 0.8 µM (0–1.4) at M24 (Figure 1A). 60–80% of the patient samples were between Cmax (therapeutic range: 3.3–11.6 µM) and Cmin (0.3–1.1 µM). The corresponding ritonavir median (range) plasma concentrations were 0.22 µM (0.08–1.1) at M0, 0.11 µM (0–1.0) at M12 and 0.22 µM (0–0.63) at M24 (Figure 1A).

The amprenavir median plasma concentrations were 2.8 µM (range: 0.7–4.2) at M0, 4 µM (3.1–5) at M12 and 4.1 µM (2.2–4.4) at M24 (data not shown). 67–80% of the patient samples were measured at Cmin (therapeutic range: 1.6–9.9 µM; Cmax, 9.9–15.8 µM). The corresponding ritonavir median plasma concentrations were 0.14 µM (range: 0–0.2) at M0, 0.16 µM (0.14–0.18) at M12 and 0.12 µM (0.11–0.13) at M24 (data not shown).

### Prelamin A was Not Detected in PBMCs from Patients being Treated with the 2NRTI+1PI/r Regimen

No prelamin A was detected by immunofluorescence staining of PBMCs obtained from any of the cohort participants at M0 (data not shown).

In order to detect alterations in prelamin A processing, PBMCs from control subjects (n = 11) and patients receiving the 2NRTI+1PI/r regimen (n = 35) were analysed by western blotting at M0 using a combination of antibodies specific to lamin A/C and prelamin A. Patient PBMCs were also analysed at M12 and M24.

Prelamin A was not detected in PBMCs from any patients regardless of PI/r, posology or plasma concentration. Representative blots from patients receiving lopinavir or atazanavir are shown in Figure 1B. Three commercial antibodies raised against various epitopes in lamins A and C showed different sensitivities for prelamin A detection. In light of these results, samples from patients exhibiting the highest lopinavir plasma concentrations were analysed using these three antibodies (Figures S2A, S2C), as well as the most sensitive commercial prelamin A specific antibody available (Figure S2B), confirming the absence of prelamin A in PBMCs from PI/r treated patients (Figure S3).

### The Protein Concentration of PI Incubation Medium Influences the Effect of PIs on Prelamin A Processing in PBMC

**Figure 2:**
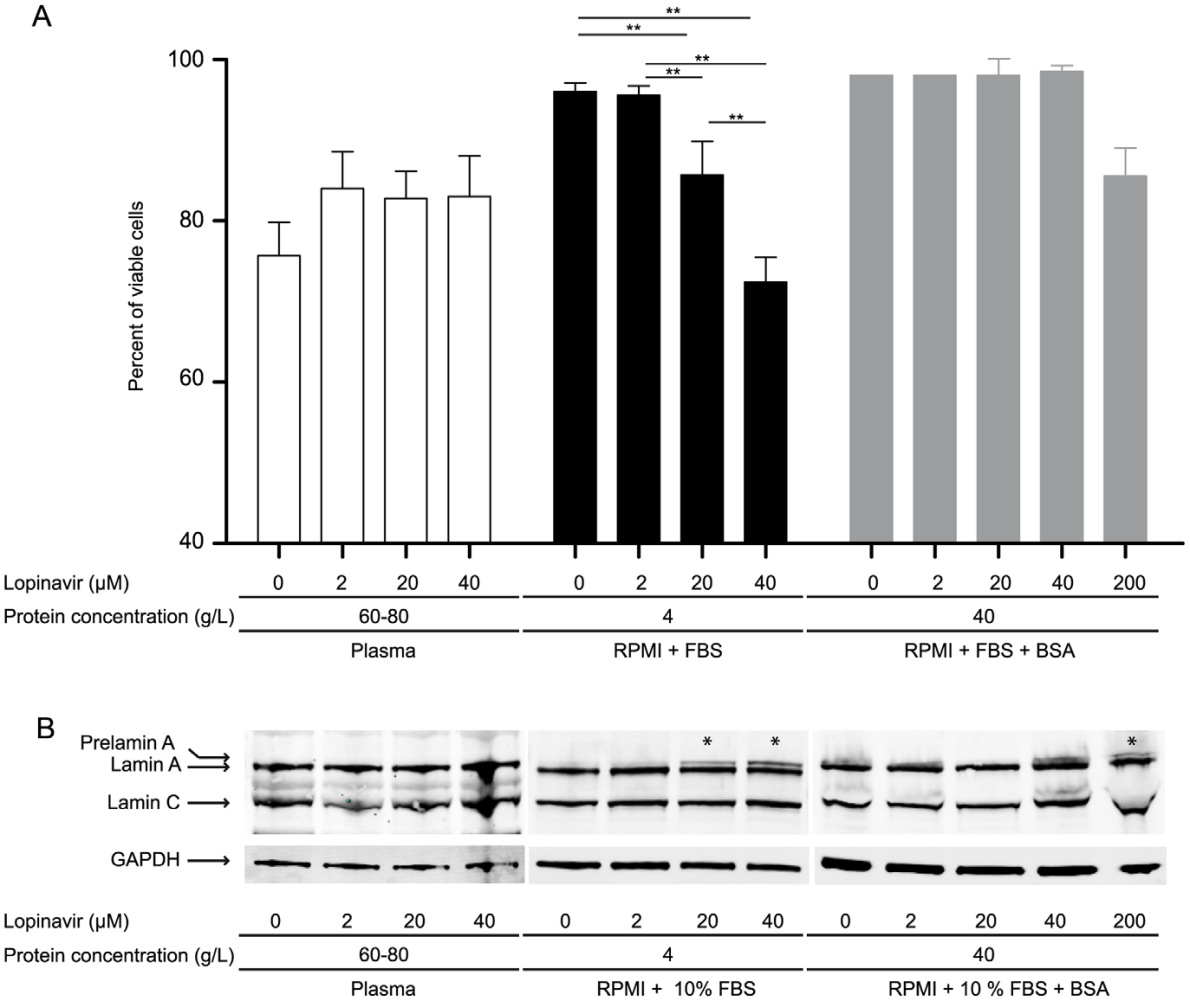
The protein concentration of PI incubation medium influences the effect of PI on prelamin A processing in PBMC. PBMC were incubated for 24 hours with increasing concentrations of lopinavir (0, 2, 20, 40, 200 µM) diluted in plasma (total protein concentration: 60–80 g/L), in RPMI culture medium supplemented with 10% FBS and 2 mM L-glutamine (total protein concentration: 4 g/L), or in the RPMI culture medium supplemented with BSA (total protein concentration: 40 g/L). (**A**) Percentage of viable cells in plasma (white), culture medium (black), and BSA supplemented culture medium (gray) (error bars = SD, n ≥3, at least 200 cells counted in each experiment). **p<0.01. No viability changes were observed when PBMC were incubated in plasma containing lopinavir. A decrease in cell viability was apparent when PBMC were incubated in culture medium containing 20 µM or 40 µM PI. Only a slight decrease in viability was observed in cells incubated in BSA supplemented culture medium containing 200 µM PI. (**B**) Western blotting experiments. Both plasma and BSA supplemented culture medium containing 2 to 40 µM lopinavir had no effect on prelamin A maturation in PBMC. Prelamin A (anti-lamin A/C H110) was detected (*) in culture medium containing 20 µM and 40 µM lopinavir, and in BSA supplemented culture medium containing 200 µM lopinavir. Fibroblasts were cultured in the presence or absence of either 20 µM lopinavir (farnesylated prelamin A positive control) or 60 µM ZoPra (unfarnesylated prelamin A positive control) for 72 hours. GAPDH was used as total cellular protein loading control. (**A**) Western blot comparing the three lamin A/C antibodies used (N18, sc6215; H110, sc20681; Jol2, MAB3211). All antibodies recognized both lamin A and lamin C. Different amounts of farnesylated prelamin A were detected by N18 and H110, as shown by the ratio of prelamin A reported to the total prelamin A+lamin A signal. In the same conditions, Jol2 did not detect prelamin A. (**B**) Western blot comparing the two prelamin A antibodies tested (sc6214, ANT0045). The sc6214 antibody exhibited a higher affinity for both farnesylated and unfarnesylated prelamin A than the ANT0045 antibody. (**C**) Prelamin A, lamin A and lamin C protein domains and antibody epitopes. Lamin A/C N18 (sc6215, green); lamin A/C H110 (sc20681, blue); lamin A/C Jol2 (MAB3211, purple); prelamin A sc6214 (pink); prelamin A ANT0045 (orange).

PBMCs from healthy donors incubated in plasma (total protein concentration: 60–80 g/L) for 24 h displayed a cell viability of 76% ±4.2 (mean ± SD) (Figure 2A). No alterations were detected after lopinavir treatment regardless of the concentration of drug used. In contrast, the viability of PBMCs incubated for the same duration in RPMI culture medium supplemented with 10% FBS (total protein concentration: 4 g/L) decreased significantly to −11% for PBMCs incubated with 20 µM lopinavir compared to untreated cells (p = 0.002); −23% for PBMCs incubated with 40 µM lopinavir compared to untreated cells (p = 0.003); and −12% for PBMC treated with 40 µM compared to 20 µM lopinavir (p = 0.001) (Figure 2A). Moreover, no loss of viability was apparent in cells incubated with 20 or 40 µM lopinavir in BSA supplemented medium (final total protein concentration: 40 g/L). Interestingly, a slight viability decrease (−12%, p = 0,07) was observed in cells treated with 200 µM lopinavir compared to cells incubated with 20 µM lopinavir (Figure 2A).

At the same time, no prelamin A was detected in PBMCs incubated in plasma regardless of the concentration of lopinavir used (Figure 2B). In contrast, prelamin A was identified in PBMC incubated in culture medium supplemented with 10% FBS and lopinavir, with the ratios of prelamin A/(prelamin A+lamin A) being 18% ±0.7% (mean ± SD, n = 3) and 30% ±5.0% (n = 3) for 20 µM or 40 µM lopinavir, respectively (Figure 2B). After the addition of BSA to the culture medium (total protein concentration: 40 g/L), no prelamin A was observed in the presence of 20 or 40 µM lopinavir. In BSA supplemented medium, prelamin A was only detected in cells treated with 200 µM lopinavir (ratio: 30%; Figure 2B). Interestingly, the presence of prelamin A seemed to be negatively correlated with cell viability.

## Discussion

The aim of the ANRS EP45 “Aging” study is to investigate the cellular mechanisms involved in the accelerated aging of HIV-1 infected and treated patients. Previous studies demonstrated that in cultured cells, PI/r induced alterations in prelamin A processing similar to those already reported in some genetic progeroid syndromes, such as progeria or restrictive dermopathy. Our hypothesis was that PI/r could reproduce the molecular mechanism underlying these genetic diseases through the accumulation of farnesylated prelamin A, thus contributing to aging in HIV infected and treated patients.

### Lopinavir Induced Prelamin A Accumulation in Cultured Cells

Several publications have previously reported the induction of prelamin A accumulation after PI treatment of various cultured human cell types, including dermal fibroblasts [31], [32], differentiating adipocytes [36], [46], HCAECs (human coronary artery endothelial cells, [34]) and osteosarcoma Saos-2 cells [33].

In these studies, the duration of PI treatment varied from 48 h up to 30 days. Interestingly, not all PI molecules seemed to induce this phenomenon. Indeed, after incubation for at least 10 days, indinavir (10–15 µM) or nelfinavir (5–15 µM) but not amprenavir (15 µM), all of which are first generation PI, induced prelamin A accumulation within cells [36], [46]. Nonotheless, despite the lack of prelamin A detection during amprenavir treatment, a slight increase in the number of dysmorphic nuclei compared to control cells has been described [46].

This disparity was also observed using second generation PIs. Indeed, incubation of cultured cells (48 h [33], 30 days [34]) with at least 10 µM lopinavir [31], [32], [33] or “boosted” lopinavir [32], [34] induced the accumulation of lamin A precursor. Atazanavir induced prelamin A accumulation at 20 µM (48 h [31], [32], [33]), but not at 4 µM (passage 2–16, [36]). Moreover, darunavir did not induce prelamin A accumulation (20 µM, 48 h, [32]).

The differential effects of PIs on lamin A processing could be linked to their different chemical structures and their different affinities for FACE1/ZMPSTE24, which have already been shown for their target HIV protease [47]. Lopinavir and atazanavir are derived from saquinavir (a first generation drug), whereas darunavir is derived from amprenavir [32].

In accordance with previous publications, we observed prelamin A accumulation in cultured dermal fibroblasts treated with lopinavir (20 µM, 72 h). In our experiments, the ratio of prelamin A to lamin A was lower (ratio = 0.08) compared to that already reported for a similar cell type and technical conditions (ratio = 0,23 [31]), which may be explained by the difference in the duration of PI treatment (72 h vs 10 days). Interestingly, this ratio was much higher (ratio = 0.26) when using an anti-lamin A/C antibody raised against a different epitope (Figure S2). This emphasised the difference in sensitivity of antibodies for prelamin A. The percentage of mature lamin A was reported as 32% after lopinavir treatment (20 µM, 48 h) in another cell type (Saos2 cells) under different experimental conditions (metabolic labelling, immunoprecipitation [33]).

We showed for the first time that prelamin A accumulation was induced in PBMCs cultured in medium containing 20 µM or 40 µM lopinavir for 24 h. The accumulation of prelamin A increased with PI concentration.

Accumulation of prelamin A induced by PIs has been shown to result from the partial inhibition of ZMSPTE24 [31], [32], [48]. PI activity was measured via the cleavage of yeast alpha mating factor by mouse Face1/Zmpste24 overexpressed in yeast cells in which the *ste24* gene, which encodes the alpha mating factor protease, was deleted. In these conditions, the half maximal inhibitory concentration (IC50) was different for lopinavir (18.4 µM [31]; 25 µM [32]) and atazanavir (150 µM [32]), suggesting that Face1/Zmpste24 was inhibited to a greater extent by lopinavir than by atazanavir [31], [32]. The IC50 of lopinavir for yeast ste24 was 125.6 µM [48]. Face1/ZmpSte24 activity remained at over 90% in the presence of darunavir or amprenavir (up to 150 µM, [31]).

Farnesylated prelamin A, resulting from inhibition of FACE1/ZMPSTE24, migrated slightly more rapidly on SDS-PAGE than unfarnesylated prelamin A (Figure S2), as previously shown [31], [32]. FACE1/ZMPSTE24 inhibition was reported to be reversible [33]. Indeed, only 3 hours after lopinavir wash out, more than 50% of prelamin A disappeared and the protein was totally undetectable after 7 hours. Nonetheless, nuclear morphology later returned (7–15 h) to a normal shape and size [33]. Furthermore, in PI exposed cells, a minor prelamin A band appeared and increased with passage number in culture, indicative of cell senescence [34].

### No Prelamin A was Detected in PBMCs from Patients being Treated with the 2NRTI+1PI/r Regimen

Our patients were considered as clinically stable regarding viral load and CD4+ cell count [38]. The mean duration of 2NRTI+1PI/r treatment was 2.7 (±1.3) years when patients were recruited at baseline (M0) [38]. Because some patients began their 2NRTI+1PI/r treatment two years before the M0 sampling, the duration of ART exposure was doubled in these patients in the course of the study. Drug plasma concentrations confirmed patient adherence to treatment and were in accordance with specific pharmacokinetic data previously reported [49]. The large majority of plasma concentrations remained within therapeutic ranges and were stable during follow-up (M0, M12 and M24).

None of the 5 patients being treated with an ART regimen including fosamprenavir displayed prelamin A in PBMCs. However, fosamprenavir, which is the prodrug of amprenavir, was not expected to induce prelamin A according to *in vitro* studies [32], [46].

Additionally, none of the 5 patients being treated with an ART regimen including atazanavir displayed prelamin A in PBMCs. These results contrasted with data reported for one patient only [34], in whom the drug plasma concentration was not measured. Nonetheless, higher drug concentrations than those recorded in our patients failed to induce prelamin A in cultured cells [36].

Interestingly, and in contrast with data showing the inhibition of Face1/Zmpste24 by lopinavir in cell culture, none of the 25 patients being treated with an ART regimen including lopinavir presented with alterated prelamin A processing in PBMC. Our observation was not in accordance with a study showing the presence of lopinavir-induced prelamin A in PBMCs from 3 patients whose lopinavir plasma concentration was unknown [34]. Nonetheless, this prelamin A absence could be directly correlated to the discrepancy we observed between the *in vitro* and *in* *vivo* models, and may be explained by the protein binding affinity of PIs and the difference in protein concentration in plasma and cell culture medium.

### Several Factors May Explain the Differential Effect of PI on Lamin A Processing in Cultured Cells and in Patient PBMC

PIs exhibit highly specific and saturable binding to proteins, particularly the plasma proteins serum albumin (SA) and alpha 1 acid glycoprotein (AAG) [50], [51], [52]. Because more than 90% of PIs are bound to plasma proteins, only low levels of the drugs remain available to be imported into cells. Nevertheless, the intracellular PIs concentration has not been directly related to protein binding *per se* [52]. Additionally, it is likely that PIs also bind to intracellular proteins [51]. In treated patients, therapeutic drug monitoring is performed by quantification of the drug concentration in plasma. This procedure is used to monitor the dose suitability, and in some cases can detect patient non-compliance with their treatment schedule. The values obtained are considered to reflect exposure to drugs and correspond to total drug concentration, including both the bound and unbound drug fractions.

*In vitro*, culture medium supplemented with 10% FBS (total protein concentration: 4 g/L) contains much less SA and AAG than human plasma (total protein concentration: 60–80 g/L), thus increasing the concentration of unbound PI available to cells [53]. Our results obtained with cells exposed to drugs incubated in plasma (total protein concentration: 60–80 g/L) or in culture medium supplemented with BSA (total protein concentration: 40 g/L) strongly suggested that this phenomenon is of importance. Indeed, both viability and lamin A processing were not altered when PI were incubated in culture medium supplemented with a high concentration of protein. In contrast, cells incubated with PI in culture medium containing a low concentration of proteins exhibited a decrease in cell viability and an accumulation of prelamin A. This is in accordance with the inhibitory effect of PI on the prelamin A processing protease, FACE1/ZMPSTE24. Moreover, even if the total PI concentration used in cultured cells is reported to be close to the maximal PI concentration assayed in the plasma of treated patients, few PI molecules are bound to proteins in culture medium, thus increasing the amount of PI available to cells. Therefore, PI concentration exceeds the drug therapeutic range.

Additionally, in cultured cells, the entry of ART drugs into cells is facilitated by the vehicle used (diluted ethanol or DMSO), which likely bypass PI membrane transporters and increase the drug concentration within cells [54].

Indeed, PIs, like other ART drugs, need to enter cells to reach their targets. These mechanisms have long been believed to involve diffusion that depends mainly on the size of the molecules, their lipophilic properties and their binding to proteins [52], [53]. However, active influx (organic anion transporters, OAT; organic cation transporters, OCT) [55] and efflux transporters (P-gp or MRP (multidrug resistance associated protein)) [56] play crucial roles in modulating the intracellular concentration of PIs. The expression of some of these transporters has been reported to be cell- or tissue-related (intestine, liver, kidney, blood brain barrier and immune cells [51], [52]). Interestingly, the PI concentration in CD8+ cells was lower than in CD4+ cells, while the latter expressed less P-gp than the former [52]. To date, comparative studies of the plasma and intracellular concentrations of PIs remain inconsistent, with some studies demonstrating a correlation between these two parameters while others did not [51], [52].

Therefore, the influx/efflux dynamics of drugs and their pharmacokinetic characteristics following oral administration, in contrast with the more direct exposure of drugs to cultured cells, are further differences between *in vitro* and *in vivo* situations. Indeed, in human patients, PIs and other drugs are constitutively metabolized by p450 cytochrome enzymes, mainly of the CYP3A subfamily in the liver [50], [51]. Furthermore, patient PI regimens, but not cell culture medium, contained ritonavir as a CYP3A4 inhibitor, thus leading to potentiation of lopinavir (the main PI in our cohort) [57]. Therefore, the effect of PI on lamin A processing was expected to be emphasised in *in vivo* compared to *in vitro* studies.

For all of these reasons, data obtained in cultured cells may be quite different from data recorded in cells from patients. This may explain the absence of prelamin A in PBMCs from PI/r treated patients. Moreover, additional discrepancies between *in vitro* and *in vivo* studies have also been described regarding PI mitochondrial toxicity. Whereas an *in vitro* study highlighted the inhibition of the mitochondrial protease processing by PIs [58], we did not confirm these data in PBMC mitochondria [38].

### Could PI be Less Toxic to PBMC than to Other Cells?

In patients, PI can accumulate to different extents in various tissues and in PBMC [59], [60], [61], [62]. Therefore, PI toxicity may be observed in some tissues but not in others.

*Ex vivo* analyses have previously been performed on adipose tissue from patients being treated with indinavir or nelfinavir regimens who presented with abnormal fat repartition (ART mean duration: 3 years, [36], [37]). The patient tissues exhibited prelamin A accumulation as well as an increased percentage of small adipocytes, fibrosis without inflammatory features, and a decrease in the number of blood vessels compared to control tissue samples [37].

A small percentage of patients from our cohort (28% receiving 2NRTI+1PI/r as well as 25% receiving 2NRTI+1NNRTI) exhibited an abnormal fat distribution. Prelamin A was not detected in PBMCs from these patients.

Interestingly, mice treated with Kaletra (lopinavir/ritonavir; 200/50 mg/kg/day) exhibited no changes in glycemia after a 2–8 week exposure, although hypertriglyceridemia was observed. Moreover, long-term treatment (8 weeks) markedly remodelled peripheral inguinal adipose deposits but did not alter epididymal fat deposits or brown adipose tissues. These observations were not reproduced with unboosted atazanavir treatment, and only partially mimicked ART-associated lipodystrophic syndromes in humans [63]. Nonetheless, and despite a circulating drug concentration within the therapeutic range expected in patients, mice showed a rapid elimination of ART drugs with a half-life shorter than in humans [63].

Some PI molecules such as indinavir and nelfinavir [46], [64] or lopinavir [63] have already been reported to induce altered expression of the adipogenic factor, SREBP1, a transcription factor known to play an important role during adipocyte differentiation. In cultured differentiating adipocytes, indinavir induced SREBP1 sequestration at the nuclear periphery [65]. Inhibition of FACE1/ZMSPTE24 by PIs has already been suggested to contribute to the development of lipodystrophy [33]. Nonetheless, the impact of prelamin A accumulation on adipocyte differentiation remains controversial [66], [67].

In cultured cells, impairment of prelamin A processing by PI has been shown to be related to the drugs used and their concentrations, thus reproducing the pathophysiological mechanism of systemic progeroid syndromes. In PBMCs from patients, PI/r first line regimens did not result in prelamin A processing abnormalities. Therefore, PI/r do not seem to be involved in PBMC lamin A-related aging. We demonstrated that lamin A processing abnormalities were observed in cultured PBMCs from healthy donors only if the protein concentration in the culture medium was low, thus increasing the amount of PI available to enter cells. Nonetheless, the effects of PI/r could be different in other cell types.

## Supporting Information

Figure S1 **PBMC incubated with ZoPra, as a positive control for the immunodetection unfarnesylated prelamin A.** PBMCs from healthy seronegative subjects were incubated for 24 hours in culture medium containing 60 µM ZoPra. Prelamin A was only detected in cells incubated with ZoPra. (**A**) Western blotting of PBMC protein extracts using a prelamin A-specific antibody and three different lamin A/C-specific antibodies. (**B**) Immunofluorescence microscopy of PBMC double labelled with antibodies against prelamin A and lamin A. Bar, 20 µm. (**C**) Flow cytometry using antibodies against prelamin A and lamin A.
(TIF)

Figure S2 **Antibody characterization.** Fibroblasts were cultured in the presence or absence of either 20 µM lopinavir (farnesylated prelamin A positive control) or 60 µM ZoPra (unfarnesylated prelamin A positive control) for 72 hours. GAPDH was used as total cellular protein loading control. (**A**) Western blot comparing the three lamin A/C antibodies used (N18, sc6215; H110, sc20681; Jol2, MAB3211). All antibodies recognized both lamin A and lamin C. Different amounts of farnesylated prelamin A were detected by N18 and H110, as shown by the ratio of prelamin A reported to the total prelamin A+lamin A signal. In the same conditions, Jol2 did not detect prelamin A. (**B**) Western blot comparing the two prelamin A antibodies tested (sc6214, ANT0045). The sc6214 antibody exhibited a higher affinity for both farnesylated and unfarnesylated prelamin A than the ANT0045 antibody. (**C**) Prelamin A, lamin A and lamin C protein domains and antibody epitopes. Lamin A/C N18 (sc6215, green); lamin A/C H110 (sc20681, blue); lamin A/C Jol2 (MAB3211, purple); prelamin A sc6214 (pink); prelamin A ANT0045 (orange).
(TIF)

Figure S3 **Prelamin A was not detected in PBMC from patients exhibiting the highest plasma concentration of lopinavir.** (**A**) Western blotting of protein extracts prepared from PBMCs isolated from healthy controls and incubated in culture medium containing 60 µM ZoPra for 24 hours. Prelamin A was only detected in cells incubated with ZoPra. (**B**) Western blotting of protein extracts prepared from PBMCs isolated from ANRS EP45 “Aging” patients exhibiting the highest plasma concentration of lopinavir (16.7–19.0 µM). The ritonavir concentration is also reported (0.7–1.8 µM). No prelamin A was detected.
(TIF)

Protocol S1 **The ANRS EP45 “Aging” study.** http://clinicaltrials.gov/, NCT01038999.
(PDF)

Methods S1 **Dermal fibroblast culture, protein extraction, immunofluorescence and western blotting procedures.**
(DOC)
