# Correction: HIV Protease Inhibitors Do Not Cause the Accumulation of Prelamin A in PBMCs from Patients Receiving First Line Therapy: The ANRS EP45 “Aging” Study

**DOI:** 10.1371/annotation/f02cdfd3-b271-46fd-a78f-5a030f0b416d

**Published:** 2013-08-14

**Authors:** Sophie Perrin, Jonathan Cremer, Olivia Faucher, Jacques Reynes, Pierre Dellamonica, Joëlle Micallef, Caroline Solas, Bruno Lacarelle, Charlotte Stretti, Elise Kaspi, Andrée Robaglia-Schlupp, Corine Nicolino-Brunet, Catherine Tamalet, Nicolas Lévy, Isabelle Poizot-Martin, Pierre Cau, Patrice Roll

The names of the twelfth and thirteenth authors were not separated by comma, the twelfth author's name was misspelled, and the two authors' affiliations were given incorrectly. The correct list of authors and their affiliations is:

Sophie Perrin^1,2^, Jonathan Cremer^1,2^¤, Olivia Faucher^3^, Jacques Reynes^4^, Pierre Dellamonica^5^, Joëlle Micallef^6^, Caroline Solas^7,8^, Bruno Lacarelle^7,8^, Charlotte Stretti^1,2^, Elise Kaspi^1,2^, Andrée Robaglia-Schlupp^1,2^, Corinne Nicolino-Brunet^9^, Catherine Tamalet^10,11^, Nicolas Lévy^1,12^, Isabelle Poizot-Martin^4^, Pierre Cau^1,2^, Patrice Roll^1,2^*

1 Inserm UMR_S 910, Marseille, France, Aix-Marseille Université, 

2 Laboratoire de Biologie Cellulaire, Centre Hospitalier Universitaire (CHU) La Timone Assistance Publique des Hôpitaux de Marseille (APHM), Marseille, France, 

3 Service d’Immuno-Hématologie Clinique, Centre Hospitalier Universitaire (CHU) Sainte Marguerite Assistance Publique des Hôpitaux de Marseille (APHM), Marseille, France, 

4 Département des Maladies Infectieuses et Tropicales, Centre Hospitalier Régional et Universitaire (CHRU) Gui-de-Chauliac, Montpellier, France, 

5 Service d’Infectiologie, Centre Hospitalier Universitaire (CHU) L’Archet 1, Sophia-Antipolis Université, Nice, France, 

6 Centre d’Investigation Clinique - Unité de Pharmacologie Clinique et d’Evaluations Thérapeutiques (CIC-UPCET), Centre Hospitalier Universitaire (CHU) La Timone Assistance Publique des Hôpitaux de Marseille (APHM), Marseille, France, 

7 Laboratoire de Pharmacocinétique et de Toxicologie, Centre Hospitalier Universitaire (CHU) La Timone Assistance Publique des Hôpitaux de Marseille (APHM), Marseille, France, 

8 Inserm UMR_S 911, Aix-Marseille Université, Marseille, France, 

9 Laboratoire d’Hématologie, Centre Hospitalier Universitaire (CHU) La Conception Assistance Publique des Hôpitaux de Marseille (APHM), Marseille, France, 

10 Fédération de Microbiologie Clinique, Centre Hospitalier Universitaire (CHU) La Timone Assistance Publique Hôpitaux de Marseille (APHM), Marseille, France, 

11 CNRS (Centre Natonal de la Recherche Scientifique) IRD (Institut de Recherche pour le development) UM 63, Aix-Marseille Université, Marseille, France, 

12 Laboratoire de Génetique Moléculaire, Centre Hospitalier Universitaire (CHU) La Timone Assistance Publique des Hôpitaux de Marseille (APHM), Marseille, France 

