# Supplementary figures and images for: HIV Protease Inhibitors Do Not Cause the Accumulation of Prelamin A in PBMCs from Patients Receiving First Line Therapy: The ANRS EP45 “Aging” Study
Source: PLoS One. 2012 Dec 28;7(12):e53035. doi: 10.1371/journal.pone.0053035 (PMC3532351; doi:10.1371/journal.pone.0053035)

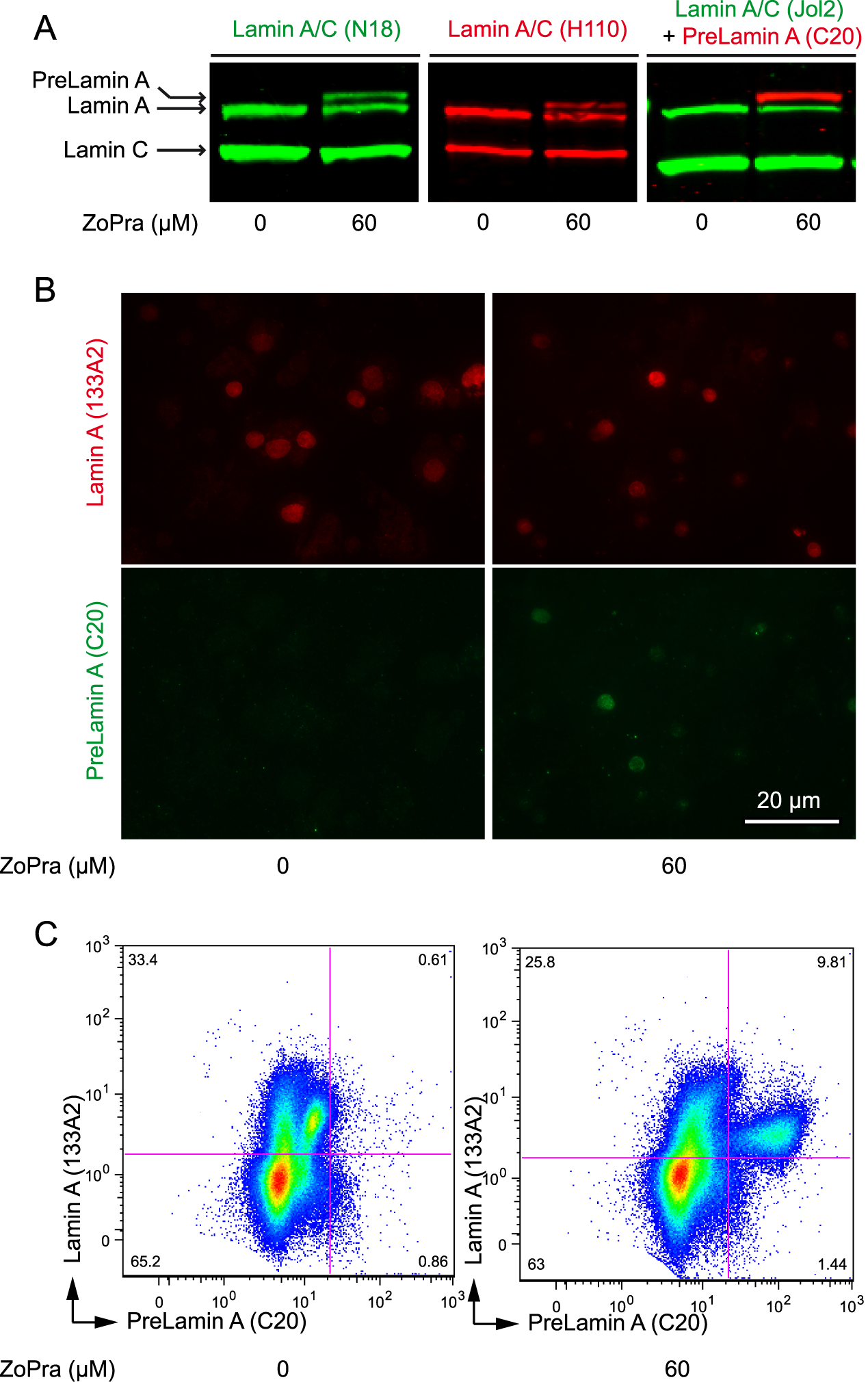

Supplement: Figure S1 — PBMC incubated with ZoPra, as a positive control for the immunodetection unfarnesylated prelamin A. PBMCs from healthy seronegative subjects were incubated for 24 hours in culture medium containing 60 µM ZoPra. Prelamin A was only detected in cells incubated with ZoPra. (A) Western blotting of PBMC protein extracts using a prelamin A-specific antibody and three different lamin A/C-specific antibodies. (B) Immunofluorescence microscopy of PBMC double labelled with antibodies against prelamin A and lamin A. Bar, 20 µm. (C) Flow cytometry using antibodies against prelamin A and lamin A. (TIF) [file pone.0053035.s001.tif]

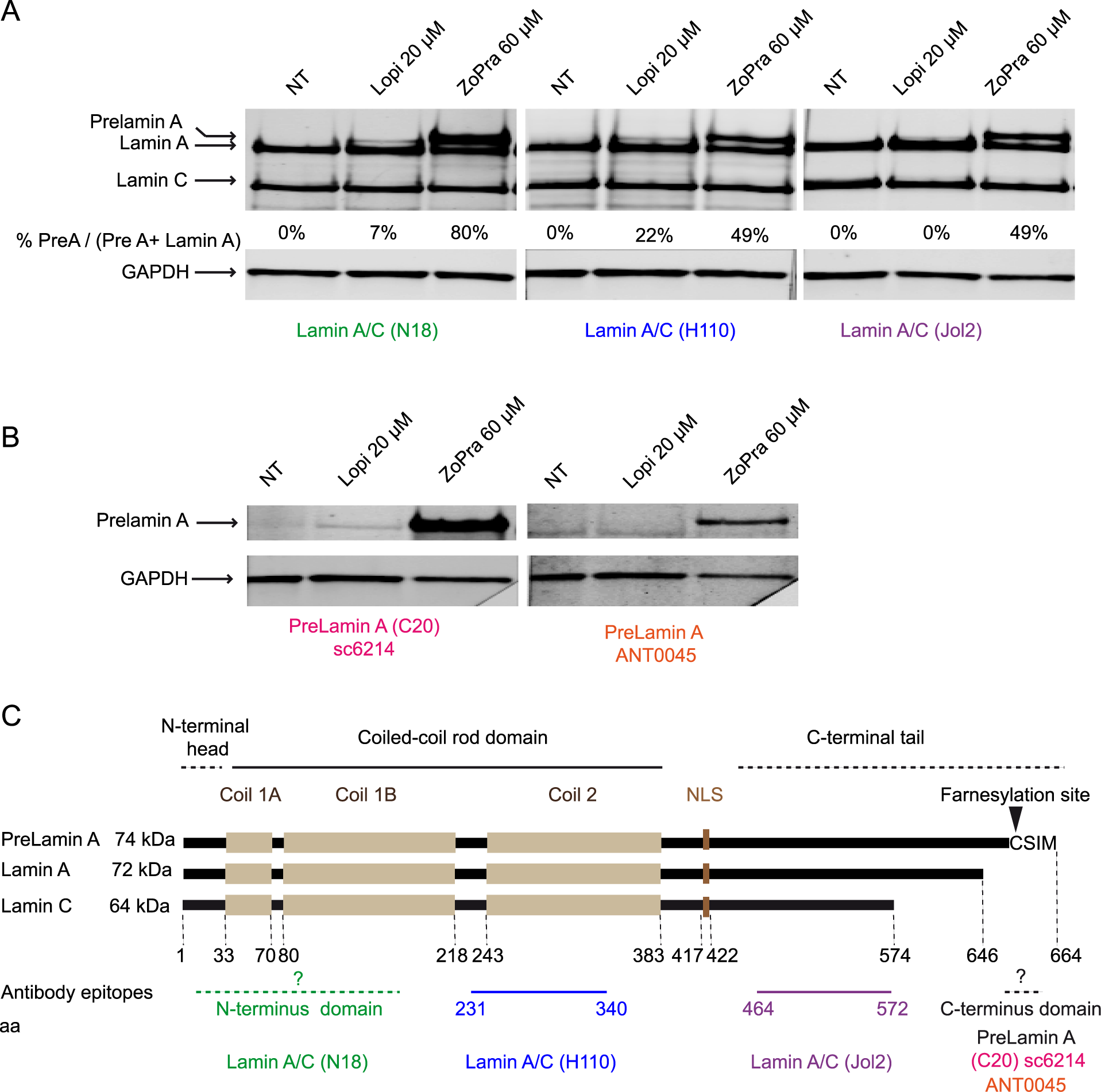

Supplement: Figure S2 — Antibody characterization. Fibroblasts were cultured in the presence or absence of either 20 µM lopinavir (farnesylated prelamin A positive control) or 60 µM ZoPra (unfarnesylated prelamin A positive control) for 72 hours. GAPDH was used as total cellular protein loading control. (A) Western blot comparing the three lamin A/C antibodies used (N18, sc6215; H110, sc20681; Jol2, MAB3211). All antibodies recognized both lamin A and lamin C. Different amounts of farnesylated prelamin A were detected by N18 and H110, as shown by the ratio of prelamin A reported to the total prelamin A+lamin A signal. In the same conditions, Jol2 did not detect prelamin A. (B) Western blot comparing the two prelamin A antibodies tested (sc6214, ANT0045). The sc6214 antibody exhibited a higher affinity for both farnesylated and unfarnesylated prelamin A than the ANT0045 antibody. (C) Prelamin A, lamin A and lamin C protein domains and antibody epitopes. Lamin A/C N18 (sc6215, green); lamin A/C H110 (sc20681, blue); lamin A/C Jol2 (MAB3211, purple); prelamin A sc6214 (pink); prelamin A ANT0045 (orange). (TIF) [file pone.0053035.s002.tif]

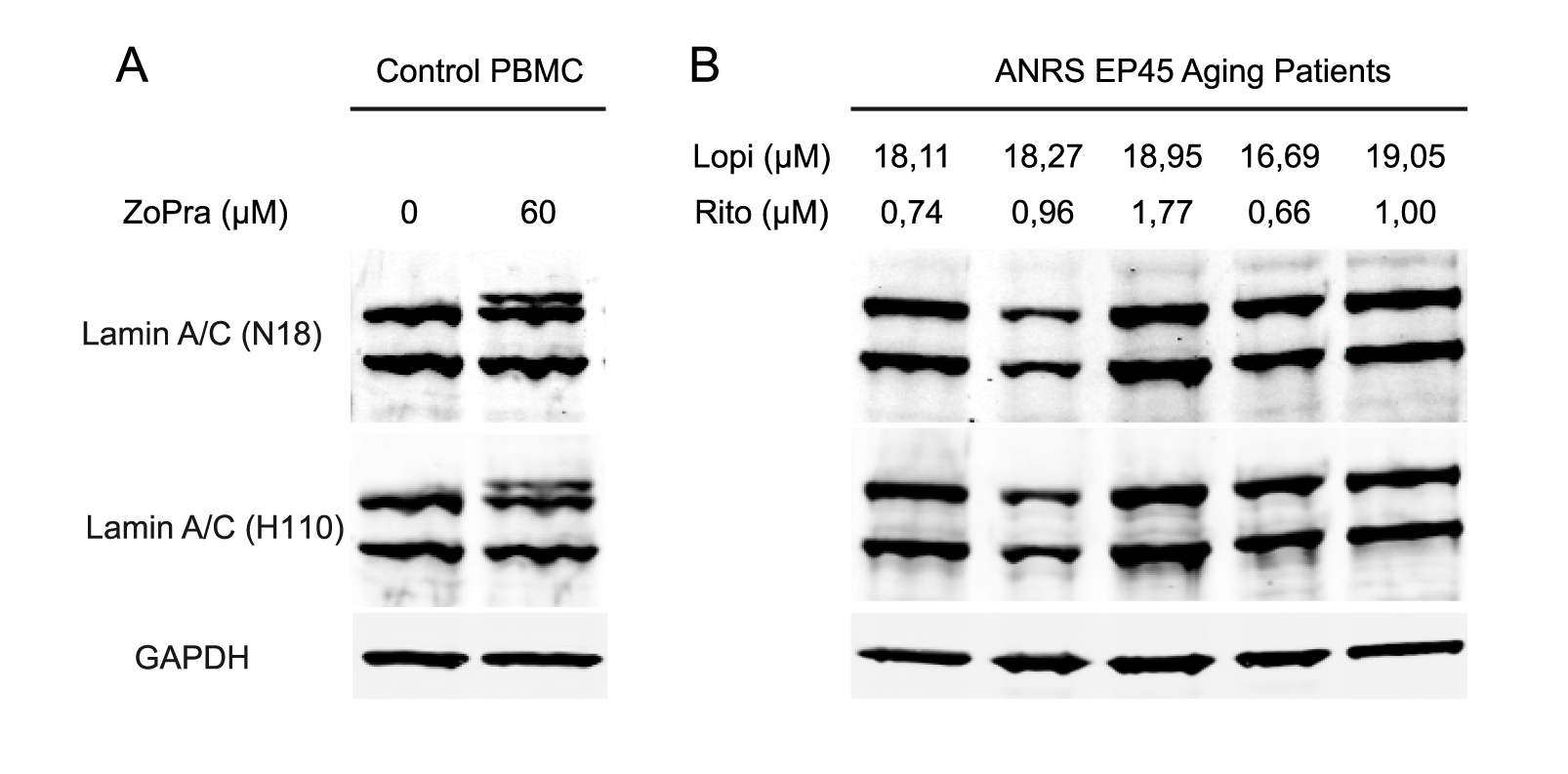

Supplement: Figure S3 — Prelamin A was not detected in PBMC from patients exhibiting the highest plasma concentration of lopinavir. (A) Western blotting of protein extracts prepared from PBMCs isolated from healthy controls and incubated in culture medium containing 60 µM ZoPra for 24 hours. Prelamin A was only detected in cells incubated with ZoPra. (B) Western blotting of protein extracts prepared from PBMCs isolated from ANRS EP45 “Aging” patients exhibiting the highest plasma concentration of lopinavir (16.7–19.0 µM). The ritonavir concentration is also reported (0.7–1.8 µM). No prelamin A was detected. (TIF) [file pone.0053035.s003.tif]
