# Supplementary material for: HIV Protease Inhibitors Do Not Cause the Accumulation of Prelamin A in PBMCs from Patients Receiving First Line Therapy: The ANRS EP45 “Aging” Study
Source: PLoS One. 2012 Dec 28;7(12):e53035. doi: 10.1371/journal.pone.0053035 (PMC3532351; doi:10.1371/journal.pone.0053035)
Supplement: Methods S1 — Dermal fibroblast culture, protein extraction, immunofluorescence and western blotting procedures. (DOC) [file pone.0053035.s005.doc]

**Supporting information**

Perrin et al.: HIV protease inhibitors do not cause the accumulation of prelamin A in PBMC from patients receiving first line therapy: the ANRS EP45 “Aging” study.

**Supplementary Methods S1:**

**Dermal fibroblast culture and drugs**

Dermal fibroblasts from healthy donors (supplied by the certified Biological Resource Center, Department of Medical Genetics (APHM Timone Hospital, Marseilles, France); from passage 7 to 10) were cultured in DMEM (Life Technologies) containing 20% FBS (Life Technologies), 2 mM L-glutamine (Life Technologies) and 2X penicillin-streptomycin (Life Technologies) at 37°C in a humidified atmosphere containing 5% CO_2_. Fibroblasts were cultured in the presence of 20 μM lopinavir or 60 μM ZoPra for 72 hours.

***Protein extractions***

Total fibroblast proteins were extracted in 300 μL extraction buffer (1% Triton X100, 0.1% SDS, 0.5% sodium deoxycholate, 150 mM NaCl, 1 mM EDTA, 20 mM Tris HCl pH 7.5, 1X protease inhibitor cocktail (Complete EDTA-free, Roche, Mannheim, Germany), 1 mM Na_3_Vo_4_, 1 mM PMSF). Cells were sonicated twice (30 sec each), incubated at 4°C for 30 minutes and then centrifuged at 10,000 g for 10 minutes. Protein concentrations were determined with the BCA™ Protein Assay (Thermo Scientific).

***Immunofluorescence***

# Directly after PBMC isolation, cells were fixed for 15 minutes at RT in 4% paraformaldehyde solution and centrifuged at 23 g for 5 minutes in a Cytospin. Slides were stored at -80°C prior to use. PBMC were permeabilized using 100 μL permeabilization buffer (0.5% Triton X-100, 50 mM NaCl, 300 mM sucrose, 20 mM HEPES pH 7.5, 3 mM MgCl_2_) for 3 minutes at RT. The permeabilized cells were incubated with the primary antibodies (mouse anti-lamin A, ab8980, 1/100, Abcam; goat anti-prelamin A, sc6214, 1/100, Santa Cruz) for 40 minutes at 37°C. After washing, the cells were then incubated with secondary antibodies (A11001, A11058, Life Technologies; 1/400) for 20 minutes at 37°C. Nuclei were stained with DAPI (0.1 μg/mL, Sigma) for 10 minutes at RT. Slides were mounted using FluorSave™ reagent (Merck). The stained cells were observed on a DM5000B/CTR5000 microscope (Leica Microsystems).

***Flow Cytometry***

Directly after isolation, PBMC were stored in RPMI containing 25% FBS and 10% DMSO at -140°C prior to further analysis.

Residual red blood cells, if any, were hypotonically lysed. Cells were fixed and washed in PBS containing 0.2 M glycine prior to being permeabilized as described above for immunofluorescence. The permeabilized cells were incubated with the primary antibodies (mouse anti-lamin A, ab8980, 1/100, Abcam; goat anti-prelamin A, sc6214, 1/100, Santa Cruz) for one hour at RT. After washing, the cells were incubated with secondary antibodies (715-116-150, Jackson Immunoresearch; A11055, Life Technologies) for one hour at RT. Mean fluorescence intensity and standard deviation (MFI ± SD) were measured by flow cytometry (Navios, Beckman Coulter Inc) on an average of 150,000 PBMC identified according to forward/side scatter (FSC/SSC). All data were analysed using FlowJo® software (Tree Star Inc).
